# The epibiotic life of the cosmopolitan diatom *Fragilariopsis doliolus* on heterotrophic ciliates in the open ocean

**DOI:** 10.1038/s41396-017-0029-1

**Published:** 2018-01-18

**Authors:** Flora J. Vincent, Sébastien Colin, Sarah Romac, Eleonora Scalco, Lucie Bittner, Yonara Garcia, Rubens M. Lopes, John R. Dolan, Adriana Zingone, Colomban de Vargas, Chris Bowler

**Affiliations:** 1 Institut de Biologie de l’École Normale Supérieure, École Normale Supérieure, Paris Sciences et Lettres Research University, CNRS UMR 8197, INSERM U1024, F-75005 Paris, France; 2 Station Biologique de Roscoff, Sorbonne Universités, UPMC Université Paris 06, CNRS, UMR7144, 29680 Roscoff, France; 3 Integrative Marine Ecology Department, Stazione Zoologica Anton Dohrn, Villa Comunale, 80121 Naples, Italy; 4 Sorbonne Universités, UPMC Univ Paris 06, Univ Antilles, Univ Nice Sophia Antipolis, CNRS, Evolution Paris Seine - Institut de Biologie Paris Seine (EPS - IBPS), 75005 Paris, France; 5 Department of Biological Oceanography, University of São Paulo, Praça do Oceanográfico 191, Cidade Universitária, São Paulo, 05508-120 Brazil; 6 Sorbonne Universités, UPMC Univ. Paris 06, CNRS, Laboratoire d’Océanographie de Villefranche, CNRS UMR 7093, Villefranche-sur-mer, Paris, 06230 France

**Keywords:** Symbiosis, Molecular ecology, Microbial ecology

## Abstract

Diatoms are a diverse and ecologically important group of phytoplankton. Although most species are considered free living, several are known to interact with other organisms within the plankton. Detailed imaging and molecular characterization of any such partnership is, however, limited, and an appraisal of the large-scale distribution and ecology of such consortia was never attempted. Here, observation of *Tara* Oceans samples from the Benguela Current led to the detection of an epibiotic association between a pennate diatom and a tintinnid ciliate. We identified the diatom as *Fragilariopsis doliolus* that possesses a unique feature to form barrel-shaped chains, associated with seven different genera of tintinnids including five previously undescribed associations. The organisms were commonly found together in the Atlantic and Pacific Ocean basins, and live observations of the interaction have been recorded for the first time. By combining confocal and scanning electron microscopy of individual consortia with the sequencing of high-resolution molecular markers, we analyzed their distribution in the global ocean, revealing morpho-genetically distinct tintinnid haplotypes and biogeographically structured diatom haplotypes. The diatom was among the most abundant in the global ocean. We show that the consortia were particularly prevalent in nutrient-replete conditions, rich in potential predators. These observations support the hypothesis of a mutualistic symbiosis, wherein diatoms acquire increased motility and tintinnids benefit from silicification through increased protection, and highlight that such associations may be more prevalent than currently appreciated.

## Introduction

Marine phytoplankton are photosynthetic microbes responsible for around half of Earth’s net primary production [1]. Diatoms, a ubiquitous and predominant component of phytoplankton, are enveloped in a characteristic silica cell wall known as the frustule, and have been proposed to contribute around 40% of marine net primary productivity [2]. They serve as the basis of the marine food web and are significant players in global biogeochemical cycles [3, 4]. Diatoms are frequently reported to dominate phytoplankton communities in well-mixed coastal, as well as upwelling regions, where light and nutrients are available [5]. They are nonetheless frequent and diverse in open ocean oligotrophic systems [6] where their survival in such low-nutrient regions can depend on mutualistic associations with other plankton. For instance, some diatoms live in obligate or facultative symbioses with heterocystous N2-fixing cyanobacteria [7]. Diazotrophic bacteria such as *Richelia intracellularis* and *Calothrix rhizosoleniae* provide nitrogen in usable forms to the diatoms (e.g., *Hemiaulus* and *Rhizosolenia* spp.), which in return may provide structural protection to the cyanobacteria.

Planktonic diatoms have been described in numerous other biological interactions, involving a range of organisms across all domains of life, as well as viruses [8]. Beyond predation, competition, and parasitism, examples include endosymbiotic diatoms in nummulitid foraminifera (e.g., *Thalassionema*-related species in the foraminifera *Heterostegina depressa* [9]), in dinoflagellates (e.g., *Galeidinium rugatum* and *Durinskia baltica* [10, 11]), and pennate diatoms with attached bacteria [12], copepods [13, 14], other diatoms (e.g., *Pseudo-nitzschia linea* and *Chaetoceros* spp. [15]), *Phaeocystis* colonies [16], flagellated stramenopiles [17], and vorticellids [18].

On the other hand, tintinnids (Choreotrichida) are heterotrophic planktonic ciliates enveloped in a species-specific test composed of organic material, the lorica [19]. They represent one of the morphologically most diverse groups of planktonic protists [20], are abundant and ubiquitous throughout the water column, with concentrations ranging from 10^1^ to 10^4^ cells per liter in surface waters [21]. A few extracellular associations between tintinnids and live diatoms have been reported, such as ones involving the radial centric diatoms *Chaetoceros* spp. and *Eutintinnus* spp. either described as “phoretic commensalism”—wherein transport is believed to be the main benefit for diatoms [22]—or suggested as a form of obligate epiphytism enabling predation avoidance for tintinnids, and access to nutrients for diatoms [23]. The chain-forming pennate diatom *Fragilariopsis doliolus* was also recorded (as *Pseudoeunotia doliolus*) with *Eutintinnus tenuis* in material collected in 10 equatorial stations between the Galapagos archipelago and the Marquesas Islands [24]. Small chains of *F. doliolus* were previously found associated with *Salpingella subconica* near the Prince Edward Islands in the Southern Ocean with rates of association involving 3–30% of all *F. doliolus* and 35–83% of *S. subconica* cells encountered, as well as in the Benguela Current [25, 26]. These authors speculate that buoyancy and protection against mesozooplankton predation are the main advantages gained by the attachment of both partners. Of a different nature is the association of *Laackmaniella* and other tintinnids with apparently empty frustules of *Fragilariopsis* and other diatoms covering their lorica, also found in the Southern Ocean, for which it has been hypothesized that the ciliates retain diatom frustules following ingestion of the cellular contents, perhaps as a means of protection through camouflage [27, 28].

The many observations summarized above tend to indicate that diatom–tintinnid associations may be more prevalent than commonly thought. However, detailed molecular and morphological characterizations of these consortia are still lacking, as is a large-scale evaluation of their biogeography and ecology. The recent *Tara* Oceans expeditions have generated a worldwide, standardized amount of multidisciplinary information focusing on open ocean and size-fractionated plankton communities in the upper layer of the ocean [29, 30]. In the current study, morphological analysis of samples collected in the Benguela Current off South Africa (Station TARA_066) led to the initial observation and isolation of an epibiotic association between a pennate diatom and a tintinnid ciliate morphologically assigned to *F. doliolus* and *Salpingella* sp., respectively. The sequencing of a specific genetic marker within the ribosomal DNA (rDNA) gene loci, the short V9 region of the 18S rDNA gene, was performed to interrogate the *Tara* Oceans V9 metabarcoding data set. This revealed the biogeography of the organisms, thus highlighting nine stations in which sequence matches of both partners were high. We further used various microscopy methods and DNA sequencing of multiple genetic markers to analyze similar and new consortia within these nine other *Tara* Oceans stations. Additionally, the V4 region was sequenced because this marker resolves more accurately diatom diversity [31], and for similar reasons sequences of the ITS loci were generated for the tintinnids [32, 33]. Overall, a continuous sequencing from the beginning of 18S to D1–28S was completed to obtain highly resolved phylogenetic information. Live recordings of the interaction confirm its true existence off the Brazilian coast, and provide insights into its ecological significance. We further provide a detailed description of the pairing specificity, biogeography, and ecology of this prevalent interaction between an ecologically important diatom and heterotrophic ciliates.

## Materials and methods

The sampling strategy used in the *Tara* Oceans expedition is described in Pesant et al. [34], and samples used for morphology and genomics are listed in Table S1.

### Morphological investigation of the consortium

#### Confocal laser-scanning microscopy (CLSM)

Consortia were analyzed from samples fixed on board *Tara* (in 2010) with a mix of formaldehyde (1% final concentration) and glutaraldehyde (0.25% final concentration) and imaged by CLSM (Leica TCS SP8), equipped with an HC PL APO 40×/1.10 W motCORR CS2 objective. Multiple fluorescent dyes were used to observe the cellular components of the ciliate and the microalgae, such as the nuclei (blue, Hoechst, Ex405/Em420-470) and the cellular membranes (green, DiOC6, Ex488/Em500-520), and cell surface (cyan, AlexaFluor 546 Ex552Em560-590). The autofluorescence of the chlorophyll was also visualized (red, Ex638/Em680-700). Sample preparation, staining, mounting, and CLSM protocols are detailed in Colin et al. [35]. Image processing and three-dimensional reconstructions were conducted with Fiji [36] and IMARIS (Bitplane) software. For consortia quantification, see Supplementary Information. Additional observations were made on samples from the Outpace Cruise in 2015; for details of sampling protocol and processing see Dolan et al. [37].

#### Scanning electron microscopy (SEM)

For SEM, we used 0.5 ml of 20–180 µm size fraction surface formaldehyde-fixed samples from Station TARA_102 placed on 3 µm pore size nucleopore filters, washed in distilled water, dehydrated in ethanol series (25, 50, 75, 95, and 100%) and critical point-dried. Dried filters were mounted on stubs, sputter-coated with gold-palladium, and observed using a JSM 6700F (JEOL Ltd, Tokyo, Japan).

#### Live recording of the consortia

Natural plankton samples were collected at a fixed station off Ubatuba, Brazil (23.52° S, 45.09° W) by means of horizontal tows of a 1.5 m long, 20 μm mesh sized net, between 21st June and 26th July 2017. Live samples were diluted with surface seawater, transferred to an insulated container, and immediately transported to the laboratory, where they were kept in a walk-in, temperature-controlled room set to match the ambient seawater temperature (21–23 °C). Upon no more than 1 h after sampling, aliquots of the diluted sample were pipetted onto a Fluorodish (WPI, Sarasota, USA) and observed under an Olympus IX73 inverted microscope equipped with a 40X objective and a Photron SA2 high speed camera. Image sequences were taken at 500 and 1000 frames per second (fps), and stored at 30 fps for visualization.

### Station selection for isolation and molecular identification of the consortia

#### Universal PCR amplification of the V4-V9 18S rDNA subregion

Single consortia composed of the *Fragilariopsis–Salpingella* associations were isolated from formaldehyde (1%)–glutaraldehyde (0.25%)–fixed surface samples collected by a microplankton net (20–180 μm mesh size) in Station TARA_066 located in the Benguela Current in 2010 (Figures S1, S2). Using a glass micropipette, consortia were collected based on morphological traits of the barrel-shaped diatom, rinsed two to three times in a minimum volume of sterile artificial seawater, before proceeding to DNA extraction, universal-eukaryotic V4-V9 PCR amplification, cloning, plasmid purification, and sequencing (see Supplementary Information).

#### Detection in *Tara* Oceans stations

Sequencing results were assembled and trimmed to retain the V9 18S fragment and interrogated against the *Tara* Oceans ribotype database using BLAST [38]. The *Tara* Oceans nucleotide sequences are available at the European Nucleotide Archive (ENA) under the project PRJEB402 and PRJEB6610. Diatom isolated V9 sequences matched at 100% identity with the ribotype “f2f8b” assigned to “Raphid-pennate_X+sp”. Tintinnid isolated V9 sequences matched with three different tintinnid ribotypes and in majority with “a7cbc” and “b61a7” assigned to “Choreotrichia_X+sp.” (see Table S2 for complete barcode identifier). The search for these four sequences in the global metabarcoding data set revealed that they were widely distributed in various size fractions [39] and were positively correlated (Spearman *rho = *0.37, *p-*value < 2.2e-16)(Figure S3a). Nine additional stations were chosen for further analysis, based on the simultaneous presence of over 30 copies of the diatom and tintinnid ribotypes (summed by assignation): Stations TARA_070, TARA_102, TARA_106, TARA_109, TARA_111, TARA_122, TARA_124, TARA_128, and TARA_139, in fraction 20–180 μm of surface samples where consortia were big enough to be identified and isolated, except TARA_128 in which we investigated the 5–20 μm fraction. Abundances of the four ribotypes are shown in Table S3 completed with an additional diatom barcode (5ecf4) later discovered in Station TARA_102.

### Molecular and phylogenetic analysis of the diatom–tintinnid consortium

#### Advanced micromanipulation for cell isolation

A new method of isolation was applied to collect *Fragilariopsis–Salpingella* consortia from ethanol-preserved samples collected by a plankton net (20 μm mesh size) in the 10 selected *Tara* Oceans stations from the Indian, Atlantic, and Pacific Oceans. By using an OLYMPUS IX51 inverted microscope equipped with an Eppendorf manual microinjector CellTram® Air, single consortia were rinsed three times in 100% ethanol Labtech wells before being treated for DNA extraction according to steps in the MasterPure DNA and RNA purification kit (Epicenter). Samples used for molecular identification are available in Table S1.

#### Targeted PCR amplification of small subunit rDNA genes, internal transcribed spacers ITS1 and ITS2, 5.8S rDNA and 28S rDNA genes

To obtain different phylogenetic ribosomal markers for both partners, initial group-specific amplifications were conducted with the Phusion High-Fidelity DNA Polymerase (Finnzymes). Group-specific primers were designed and inspired from Bachy et al. [32] and McDonald et al. [40] and are shown in Table S4. For amplification protocol, see Supplementary Information. Amplicon sequences of both the diatom and the tintinnid were cleaned, trimmed, and assembled using Sequencher (version 5.4) and accession numbers are available in Table S1. Summaries of available imaging and molecular data for each station and consortia are available in Tables 1 and 2.

**Table 1:** Summary table of available information for each investigated station

Sampling station	Ocean province	Latitude	Longitude	Isolation under bright-field microscopy^a^	Single consortia genomic analyses	SEM	CLSM	Formol/lugol	Tintinnid genera assigned morphologically
TARA_066	South Atlantic Ocean	−34.8986	18.03	20 consortia	6	Yes	Yes	Yes	*Salpingella, Amphorellopsis, Amphorides, Dictyocysta*
TARA_070	South Atlantic Ocean	−20.3943	−3.2085	1 consortium	1		Yes	Yes	*Salpingella*
TARA_102	South Pacific Ocean	−5.2514	−85.1696	8 consortia	5	Yes	Yes	Yes	*Salpingella*
TARA_106	South Pacific Ocean	−0.0141	−84.5899	7 consortia	4		Yes	Yes	*Salpingella, Amphorellopsis*
TARA_109	South Pacific Ocean	1.9958	−84.576				Yes	Yes	*Salpingella*
TARA_111	South Pacific Ocean	−16.9581	−100.6588	5 consortia					*Salpingella*
TARA_122	South Pacific Ocean	−9.006	−139.206	6 consortia	2		Yes		*Salpingella, Eutintinnus*
TARA_124	South Pacific Ocean	−9.1463	−140.515	14 consortia	6		Yes	Yes	*Salpingella, Eutintinnus, Amphorellopsis, Protorhabdonella*
TARA_128	South Pacific Ocean	0.008	−153.7022	2 consortia			Yes	Yes	*Salpingella*
TARA_139	North Pacific Ocean	6.48	−95.0153	12 consortia			Yes	Yes	*Salpingella, Eutintinnus*

Each station in which the diatom–tintinnid association was searched for carries visual evidence for its presence that is reported in this table. Morphological evidence was obtained either in ethanol fixed samples observed under bright-field microscopy from which specimens were isolated for sequencing, in scanning electron microscopy (SEM) samples, or by confocal laser-scanning microscopy (CLSM) from which the consortia was quantified, or in formol-fixed samples from which *Salpingella* spp. were distinguished. From these visual observations, six different tintinnid genera were identified as interacting with the same pennate diatom

^a^Only Salpingella consortia

**Table 2:** Summary table of available molecular information for each isolated consortia and geographic origin

Station	Latitude	Longitude	Ocean province	TI_ID	V9^a^	Diatom V9	Tintinnid V9	Diatom contigs	Tintinnid contigs
TARA_066	−34.8986	18.03	South Atlantic Ocean	882	*D*, T	No match	b61a7	KY782381	KY782355
TARA_066	−34.8986	18.03	South Atlantic Ocean	884	D, T	f2f8b	a7cbc	KY782383	KY782356
TARA_066	−34.8986	18.03	South Atlantic Ocean	886	D, T	f2f8b	a7cbc	KY782384	KY782357
TARA_066	−34.8986	18.03	South Atlantic Ocean	887	D, T	f2f8b	a7cbc	KY782385	KY782358
TARA_066	−34.8986	18.03	South Atlantic Ocean	888	*D*, T	*No match*	deb2a	KY782386	KY782359
TARA_066	−34.8986	18.03	South Atlantic Ocean	890	D, T	f2f8b^b^	a7cbc	KY782387	KY782361
TARA_070	−20.3943	−3.2085	South Atlantic Ocean	881	*D*, T	No match	No match	KY782380	KY782353
TARA_102	−5.2514	−85.1696	South Pacific Ocean	819	T		a7cbc		KY782343
TARA_102	−5.2514	−85.1696	South Pacific Ocean	820	D, T	f2f8b	a7cbc	KY782369	KY782344
TARA_102	−5.2514	−85.1696	South Pacific Ocean	821	D, T	53cf4	a7cbc	KY782370	KY782345
TARA_102	−5.2514	−85.1696	South Pacific Ocean	823	D, T	f2f8b	a7cbc	KY782371	KY782346
TARA_102	−5.2514	−85.1696	South Pacific Ocean	825	*D*	No match		KY782372	
TARA_106	−0.0141	−84.5899	South Pacific Ocean	851	D, T	No match	b61a7	KY782373	KY782348
TARA_106	−0.0141	−84.5899	South Pacific Ocean	852	D, T	f2f8b	b61a7	KY782374	KY782349
TARA_106	−0.0141	−84.5899	South Pacific Ocean	853					KY782350
TARA_106	−0.0141	−84.5899	South Pacific Ocean	854	*D*, T	No match	a7cbc	KY782375	KY782351
TARA_122	−9.006	−139.206	South Pacific Ocean	863	D, T	f2f8b	b61a7	KY782377	KY782352
TARA_122	−9.006	−139.206	South Pacific Ocean	864				KY782378	
TARA_124	−9.1463	−140.515	South Pacific Ocean	807	D	f2f8b		KY782362	
TARA_124	−9.1463	−140.515	South Pacific Ocean	808	D	f2f8b^b^		KY782363	
TARA_124	−9.1463	−140.515	South Pacific Ocean	811	*D*	No match		KY782365	
TARA_124	−9.1463	−140.515	South Pacific Ocean	813	D	f2f8b		KY782366	
TARA_124	−9.1463	−140.515	South Pacific Ocean	814	D, T	f2f8b		KY782367	KY782340
TARA_124	−9.1463	−140.515	South Pacific Ocean	815	T		b61a7		KY782341

Each isolated consortium has a unique identifier starting with “TI_8##”. For single cell barcoding, we indicate whether complete or partial (italic) V9 sequences were obtained for the D or the T and searched for the equivalent matching metabarcode sequence in *Tara* Oceans with 100% identity. Genbank accession numbers indicate contigs of sequenced molecular markers (partial 18S, ITS1 and 2, 5.8S or partial 28S)

^a^ *D* diatom, *T* tintinnid; in italic if partial

^b^ 99% identity with f2f8b

#### Phylogenetic analysis

For the tintinnids, contigs of the amplicons were obtained, and two matrices of 18S rDNA and ITS+5.8S+28S rDNA were built, including reference and outgroup sequences from Bachy et al. [32]. For diatoms, similar matrices were built including reference sequences from BLAST top hits in GenBank and reference sequences from Theriot et al. [41]. Sequences were aligned using MAFFT version 7 [42] and trimmed with Gblocks [43] before applying JmodelTest [44] to determine the best model of nucleotide substitution for each matrix. The general time-reversible model with gamma distribution of rate variation (GTR+G) was selected for the diatom ITS+5.8S+28S tree and the tintinnid 18S tree, which are, respectively, the best markers to anchor species at the genus level. Phylogenetic and Bayesian inferences were performed using PhyML 3.0 [45] and the program MrBayes [46], respectively (see supplementary information). Trees were visualized and edited using FigTree v.1.4.2. For ITS+5.8S+28S rDNA sequences, a statistical parsimony network was constructed and visualized with TCS software [47]. All alignments used for phylo-genetic inference are available in Table S5.

### Environmental and community contextualization of the interaction

#### Partial least square analysis

We investigated correlations between environmental parameters collected during *Tara* Oceans (doi: 10.1594/PANGAEA.853810) and the read abundance of the two partners. As variables were shown to be multicolinear, partial least square regression analysis was conducted on range-transformed oceanographic data as predictors and Hellinger-transformed read abundance data of corresponding barcodes as responses. Samples from all size fractions in surface depth were selected to increase statistical signal. Analysis was performed using the « plsdepot » package in R version 3.3.0. Predictors and responses used in the analysis as well as resulting regression coefficients are available in Table S6.

## Results and Discussion

### Discovery of a widespread diatom–tintinnid interaction

**Fig. 1:**
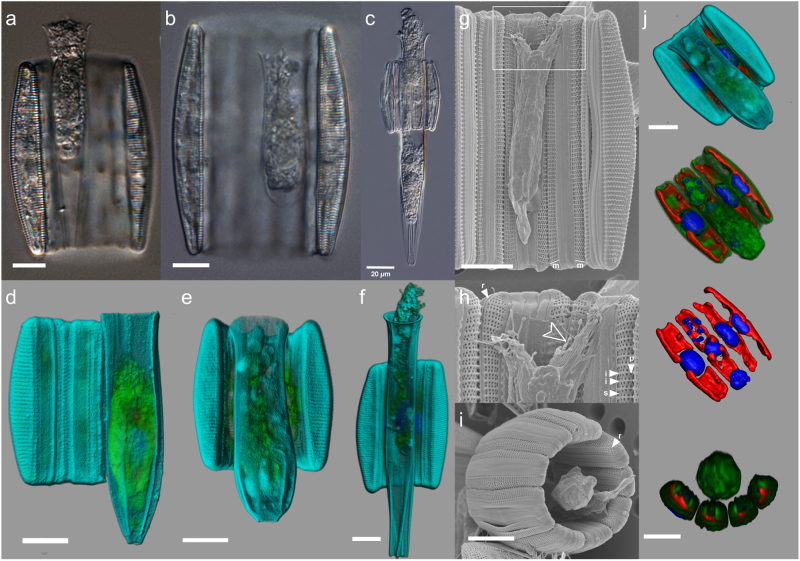
Light, electron, and confocal laser-scanning microscopy observations of *Fragilariopsis–Salpingella* associations and interface. **a, b, d, e** *Salpingella faurei* at Station TARA_066. **c** Typical smooth lorica and trumpet shaped oral opening of *Salpingella decurtata* at Station TARA_139. **d** *Amphorides laackmanni* at Station TARA_106. **f** *Salpingella curta* at Station TARA_066. **g**-**i** SEM pictures of a diatom–tintinnid consortium from Station TARA_102 **g-h**. Panel **h** is a close up view of the contact zone in **g** where the arrow indicates membranelles. The diatom frustule valves display the specific features from *Fragilariopsis doliolus*: transverse striae (s) with two alternating rows of poroids (p) at the base of the interstriae (i), the eccentric raphe system (r), lying at the junction of valve face and proximal mantle (m). Panel **i** shows a full barrel of diatom chain taken at Station TARA_102. **j** Three-dimensional (3D) reconstruction of the epibiosis involving *Salpingella faurei* imaged with confocal laser-scanning microscopy at Station TARA_066 labeling DNA (Hoechst, blue), chloroplasts (chlorophyll autofluorescence, red), membranes (DiOC6, green), cell surface (AlexaFluor 546, cyan). Scale bar = 10 μm except in **c** where scale bar = 20 μm

Confocal microscopy analysis of microplankton from *Tara* Oceans Station TARA_066 in the Benguela Current (off South Africa) revealed an interaction between a diatom and a tintinnid (Figs. 1a, b). Isolation of individual consortia by micromanipulation led to the identification of the 18S rDNA sequences of both partners, including the V9 hypervariable region (Materials and methods section). A search for the same V9–18S rDNA sequences in the *Tara* Oceans global metabarcoding data set from 126 sampling stations [39] revealed nine additional stations with high sequence abundance of both partners, which were therefore chosen for further investigation. Visual evidence of *Fragilariopsis*–*Salpingella* consortia was established in all the selected samples along with the discovery of new ciliate genera involved in the association (Table 1).

### Morphological diversity of diatom–tintinnid consortia

**Fig. 2:**
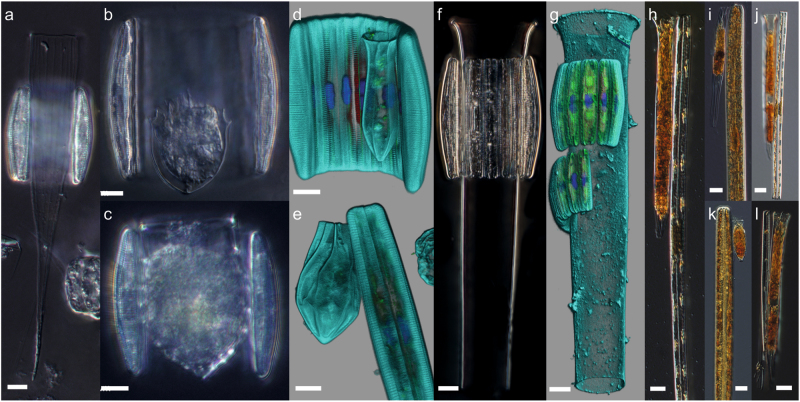
High diversity of pennate–tintinnid interactions in the open ocean observed by light microscopy and CLSM. **a** *Protorhabdonella* tintinnid at Station TARA_124. **b** *Ascampbelliella* tintinnid at Station TARA_110. **c** *Dictyocysta* tintinnid at Station TARA_066. **d** *Amphorellopsis* tintinnid at Station TARA_066. **e** Unknown tintinnid at Station TARA_066. **f, g** *Eutintinnus* tintinnid at Station TARA_124 with **f** one or **g** two diatom chains. **h-l** Tintinnid interactions with other diatom species (**h, j** and **l** probably Thalassionemataceae) extracted from the Outpace cruise (date: 28/03/2015; Lat: 18°25,15’S; Long: 165°56,38’W between New Caledonia and Tahiti). Scale bar = 10 μm

Diatom-associated ciliates displayed diverse morphologies, with at least seven different ciliate genera including five that had never been described with *F. doliolus* before (see Supplementary Information taxonomic details). Light microscopy analysis of specimens from the Benguela Current (Figs. 1a, b, d, e) were identified as *Salpingella faurei* whereas those from the tropical North Pacific Ocean (Fig. 1c) were identified as *Salpingella decurtata*. The diatom was also associated with *Salpingella curta* in the tropical southeastern Pacific Ocean (Station TARA_102, data not shown) and *Eutintinnus* spp. in the Marquesas Islands area (Fig. 2). New associations involving five ciliate genera (Figs. 2a–e) are reported here for the first time and in some cases consortia have been seen with pennate diatoms other than *F. doliolus* (Figs. 2h–l). Regarding morphology of the diatom, SEM revealed barrel-shaped chains and diatom frustule valves displaying transverse striae with two alternating rows of poroids at the base of the interstriae, the latter rising externally above the level of the valve, as well as a strongly eccentric raphe system, lying at the junction of the valve face and proximal mantle (Fig. 1g). These ultrastructural features, along with the asymmetric valve shape, clearly distinguish the pennate diatom *F. doliolus* from congeneric species, which are all symmetrical bilaterally and have smooth external valve faces [48, 49].

Several features of the consortia are noteworthy. Contrary to other cases of diatom–tintinnid associations in which lorica are generally coated with empty diatom frustules, the diatom cell content was clearly intact (e.g., Fig. 1j). Tintinnid cells were also observed within the lorica, with rare exceptions including *Eutintinnus* and *Protorhabdonella* specimens, in which it was most likely the fixative (lugol, formol, or glutaraldehyde-paraformaldehyde) that induced cell loss of the ciliate, as intact cells were repeatedly observed in ethanol-preserved samples. Colonies of *F. doliolus* differed in cell number, from a few adjacent cells surrounding less than half of the tintinnid (Fig. 1d) to a nearly complete barrel (Fig. 1i) totally hiding the ciliate. Moreover, we differentiated cases in which the *Salpingella* lorica opening was located just at the end of the diatom cell (Figs. 1b, d, e, g, h, i, j), or not (Figs. 1a, c, f), independently of the diatom–tintinnid size ratio. At times, two diatom colonies were observed on the same *Eutintinnus* cell (Fig. 2g). No tintinnids were ever found associated with an entirely empty frustule chain of *F. doliolus*. Three-dimensional reconstructions from CLSM images also showed a tight adherence of both lorica and frustule with no evidence of any specific attachment structure (Figure S1).

### Live recording of diatom–tintinnid consortia in natural coastal waters

Live recordings of the consortium from fresh samples illustrate the consortium’s mobility and occurrence off the Brazilian coast. The tintinnid motility profits both partners and enables forward (Video S1) and backward (Video S2) swimming, as well as changing directions when the ciliate membranelles encounter other organisms such as the dinoflagellate *Ceratium* (Video S3). The diatom serves as an external physical envelope that enables the tintinnid to sense co-occurring species (such as a centric diatom in Video S4) before direct contact with the lorica. A further hypothesis is that attached ciliates profit from changes in fluid dynamics of the feeding current, the so-called anchor effect, which leads to steeper velocity gradients and higher flow rates close to the lorica [50]. Moreover, even though *F. doliolus* cells possess a raphe, it may not be useful without a solid surface, so the diatom may benefit from the interaction by increased motility. As no attachment structure has been observed, this reinforces the idea that a strong biofilm composed of extracellular polymeric substances may seal both organisms. These are, to our knowledge, the first live recordings of such an association.

### Molecular identification of the interacting partners

A total of 24 individual diatom–tintinnid consortia were isolated by micromanipulation from the 10 *Tara* Oceans stations, representing a range of oceanic regions (Table 1 and Materials and methods section). We focused on *Salpingella* sp. to test for genetic specificity because it occurred in all the chosen stations. Following DNA extraction, group-specific PCR amplification was performed to sequence a series of rDNA molecular markers (18S, ITS1+2, 5.8S, 28S-D1) from both the diatom and the tintinnid in each consortium. Sequences were obtained for at least one of the partners, yielding sequences for 21 diatoms and 18 tintinnids, and we could genetically identify both partners for 15 different consortia originating from six stations (Table 2).

**Fig. 3:**
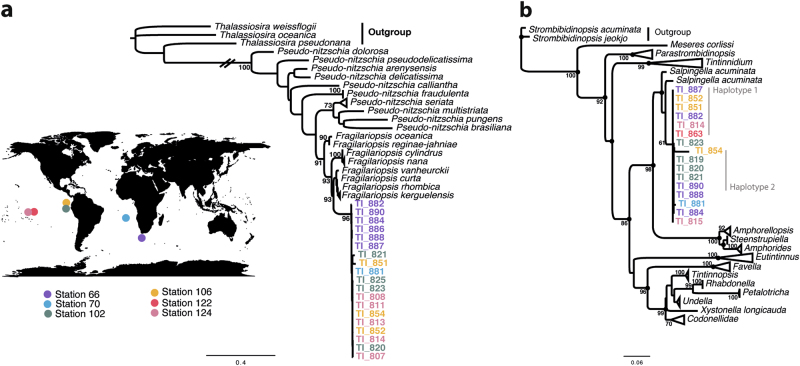
Phylogenetic identification of the diatom and tintinnid partners. **a** Diatom tree. Maximum likelihood rooted phylogenetic tree of diatom ITS + 5.8 S + 28 SrDNA sequences (854 aligned positions). Reference sequences were extracted from top assigned BLAST hits in NCBI. Numbers at nodes are percentage bootstrap values (1000X). The branch rooting of the tree has been shortened to increase clarity, and isolates are colored according to their respective stations of origin. **b** Tintinnid tree. Maximum likelihood rooted phylogenetic tree of choreotrich SSU-rDNA sequence, based on 1455 aligned positions. Reference sequences were extracted from [20, 32]. Numbers at nodes are percentage bootstrap values (1000X) (values < 70% are omitted). Bayesian posterior probabilities higher than 0.90 are indicated by filled circles. Symbiotic tintinnid isolates are colored according to their respective stations of origin, indicated in the map in panel **a**

ITS, 5.8S, and 28S molecular markers were chosen to perform the diatom phylogeny because the 18S rDNA has been considered a relatively poor marker for studies of *Fragilariopsis* and *Pseudo-nitzschia* phylogeny (but see Lim et al. [51]). The diatom sequences of the isolated specimens formed a monophyletic group (bootstrap value 96%) branching within the *Fragilariopsis* genus (bootstrap value 91%) (Fig. 3a). *Fragilariopsis* appears as monophyletic and branched within the paraphyletic *Pseudo-nitzschia* genus (Fig. 3a). These first publicly available sequences of *F. doliolus* (identified by our morphological analysis) obtained from field material revealed that the species is distinct from the previously known clades in the genus *Fragilariopsis*, which reflects the unique above-mentioned morphological characteristics of the species, as well as its much wider and more temperate distribution compared with its congeners [6].

Molecular phylogeny of the isolated tintinnid 18S rDNA confirmed the identification of *Salpingella* genus, as all *Salpingella* sequences (ours and the one previously published) grouped in a monophyletic clade with strong support (bootstrap value 97.6%; posterior probability 1). In spite of the morphological differences of the isolated specimens, we could not distinguish *Salpingella* species at the genetic level (bootstrap value 67%), indicating the limited resolution of the available 18S sequence information or illustrating a morphological plasticity of the *Salpingella* species (Fig. 3b).

**Fig. 4:**
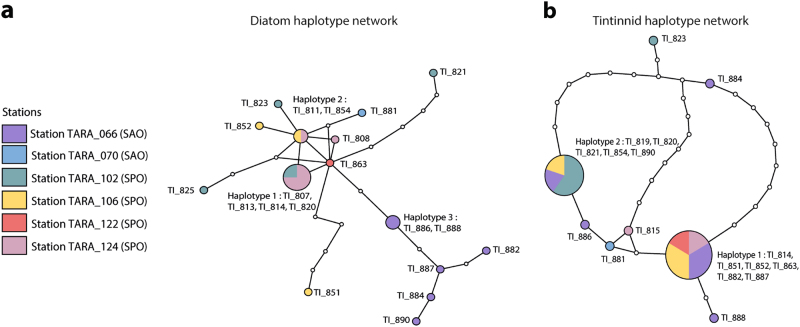
Phylogeographic patterns of both partners based on haplotype similarity networks. Sequences are from ITS+5.8S+28S rDNA. **a** Haplotypes from the diatom *Fragilariopsis doliolus* displayed more genetic diversity and biogeographical structuring particularly for sequences originating from the Benguela Current, such as from Station TARA_066 (stations and color codes are indicated in panel **a**). **b** The tintinnid *Salpingella* sp. haplotype network displays two main clusters (haplotypes 1 and 2) of sequences coming from diverse ocean regions, but with low genetic diversity overall. In each network, a white node represents one mutation, and the size of the colored circles are proportional to the abundances of sequences within a haplotype. Haplotypes are colored by the stations of origin of sequences composing it, and labeled by the “TI” identifiers. Ocean Province: SAO (South Atlantic Ocean), SPO (South Pacific Ocean)

To gain further insights into the genetic diversity of the species involved in these consortia, statistical parsimony networks of the ITS1+2 regions, together with 5.8S and the D1 region of 28S were undertaken. *F. doliolus* sequences diverged up to nine nucleotides over a 727-bp alignment using 20 sequences (Fig. 4a). Three major haplotypes were distinguished, with haplotypes 1 and 2 being composed of sequences originating from stations belonging to distant provinces. Sequences originating from the Benguela Current (Station TARA_066) all grouped together compared with the rest of the sequences and were closest to haplotype 3, showing the existence of region-specific genetic variants of *F. doliolus*.

The tintinnid network based on 17 sequences of 721 bp revealed two major haplotypes, composed of sequences from geographically distant stations (Fig. 4b). Both haplotypes can be found simultaneously in two distant stations (Station TARA_066 and TARA_106). Moreover, haplotype 1 corresponded to isolated tintinnids with a long lorica under light microscopy (similar to Figs. 1c, f), whereas haplotype 2 generally corresponded to a shorter morphotype (similar to Figs. 1a, b). This reveals that the morphological similarity of haplotypes within the isolated tintinnids is not a function of geographical separation. However, lorica length is known to be a variable character [52]. Besides TI_823 and TI_884, the other tintinnid sequences diverged from the major haplotypes by fewer than five nucleotides. The combinations of tintinnid haplotypes with *F. doliolus* haplotypes were not specific.

We further explored the genetic diversity of both partners of the consortia by focusing on the V9 subregion (Table S5), a marker widely used in metabarcoding surveys [53]. The complete V9 diatom sequences displayed three variable positions out of 130 bp with respect to other *Fragilariopsis* sequences available at NCBI, in positions 58, 70, and 86. *Fragilariopsis* species therefore display a 98% similiarity for this molecular marker. A typical 97% identity clustering of OTUs from a metabarcoding survey would have grouped together V9 sequences from *F. doliolus* and *F. curta* or *F. cylindrus*, yet none of the latter two species could possibly interact with tintinnids the way *F. doliolus* does due to the unique barrel-shape morphology of its chain. The V9 tintinnid sequences displayed one significantly variable site at position 55 and the sequences displayed a 3-bp difference with the V9 sequence available at NCBI of a *S. acuminata* isolate FG304. Additionally, tintinnids belonging to haplotype 1 (except TI_887) displayed a T at position 55, whereas tintinnids belonging to haplotype 2 displayed a C, reinforcing the existence of two haplotypes revealed with the parsimony network.

### Geographic distribution and ecological context of the consortia

**Fig. 5:**
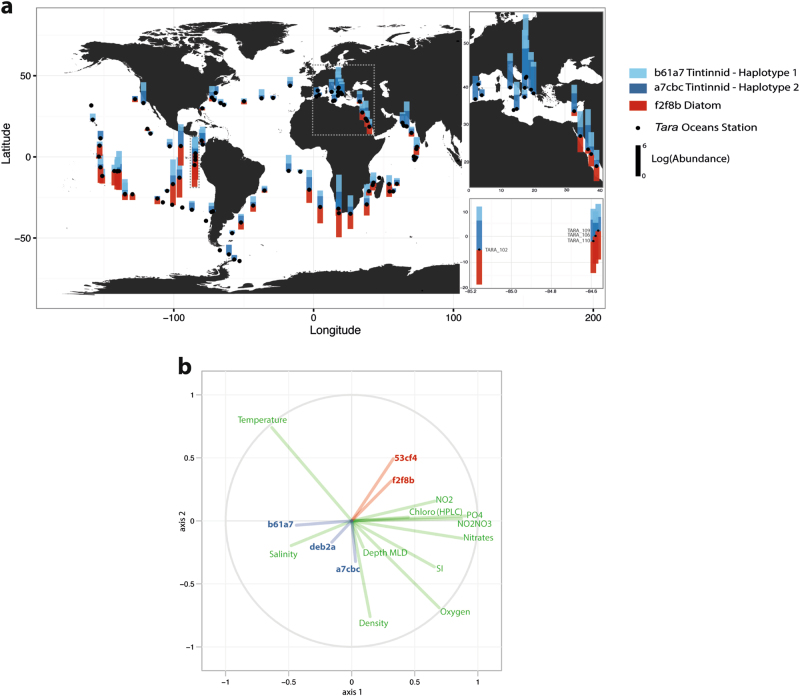
Abundance of the symbiotic diatom and tintinnid V9 rDNA sequences across the 126 *Tara* Oceans stations and environmental predictors of each symbiotic partner. **a** Absolute abundance of the most prevalent V9 rDNA sequences from the symbiotic tintinnid (a7cbc and b61a7) and diatom (f2f8b) in the *Tara* Oceans global metabarcoding data set [39]. Abundances were extracted from samples of microplankton (20–180 μm size fraction) collected in sub-surface waters [34]. Regions framed with a gray dashed line in the Mediterranean Sea, Red Sea, and South Pacific Ocean have been magnified on the right for more clarification. Absolute abundances were transformed according to the log(Abundance+1) formula. **b** Circle of correlations. The abundance of the diatom (red) and tintinnid (blue) V9 rDNA barcodes were predicted using the *Tara* Oceans metabarcoding and environmental parameters (green) data sets from surface water samples. The most prevalent tintinnid barcode a7cbc is more abundant in high density samples, whereas the main abiotic driver for the second tintinnid barcode b61a7 is chlorophyll. Regression coefficients are available in Table S6

Among the 15 complete *F. doliolus* V9 sequences obtained, 11 matched the *Tara* Oceans metabarcode f2f8b (Table 2). Significantly, barcode f2f8b, which had been assigned to “Raphid_pennate_X+sp*.”*, was among the most abundant unassigned diatom barcodes in the primary *Tara* Oceans data set, with 301,093 reads over the 293 samples from 46 sampling sites within the micro- and meso-plankton size fractions (containing organisms between 20–180 μm and 180–2000 μm, respectively) [6], representing 24.5% of the abundance of all the “Raphid-pennate_X+sp.” sequences in the top 100 unassigned barcodes. This single barcode was nearly as abundant as all barcodes assigned to the *Pseudo-nitzschia* genus (down to 80% of sequence identity), which represents the 7th most abundant diatom genus with 305,115 reads, based on Malviya et al. [6]. In light of the extended *Tara* Oceans data set [39], encompassing 126 *Tara* Oceans stations, f2f8b is the second most prevalent *Fragilariopsis* sequence with a total abundance of 414,113 reads. It is found throughout all *Tara* Oceans stations, with higher abundances reported in the southern hemisphere (0°S–40°S) and occurrences both in coastal and open ocean stations (Fig. 5a). By comparison, one diatom V9 matched at 100% identity the barcode “53cf4” that totaled 853 reads across the whole *Tara* Oceans metabarcoding data set and displayed a distribution similar to that of f2f8b (Figure S3b). The other three complete V9 sequences from isolated diatom cells were not found in the *Tara* Oceans metabarcode data set. It is possible that the V9 microdiversity was not captured in the *Tara* Oceans data due to sequencing biases or filtering of the metabarcode database, which was performed by removal of metabarcodes present in less than three reads and two distinct samples [38].

In total, the tintinnid V9 sequences matched at 100% identity to three unique barcodes in the *Tara* Oceans data set: a7cbc, b61a7, and deb2a, all of which had been assigned to “Choreotrichia_XX+sp”. Barcode a7cbc was particularily abundant and widely distributed, with an abundance of 106,292 reads over the complete *Tara* Oceans metabarcoding data set (Fig. 5a). B61a7 had a total abundance of 24,839 reads and deb2a of 3660 reads, the latter being restricted to the Cape Agulhas region (Fig. 5a and Figure S3c). All the tintinnids belonging to haplotype 1 displayed a V9 sequence corresponding to b61a7, whereas all the tintinnids belonging to haplotype 2 displayed a V9 corresponding to a7cbc. The pairing of the V9 sequences belonging to the two partners within the consortium was not specific, coherent with the conclusions of the haplotype combinations (Table S4).

Sequences with 100% identity to the specific diatom and tintinnid ribotypes were extracted from all the size fractions from surface samples of the 126 *Tara* Oceans stations and analyzed with respect to oceanographic variables such as temperature, nitrate, phosphate, and silicate through partial least square analysis (Fig. 5b). F2f8b and 53cf4 diatom barcode abundances were explained by temperature (regression coefficients of 0.21 and 0.02, respectively), nitrate (0.23 and 0.01), and density (−0.25 and −0.02), a measure derived from temperature and salinity. Abundance of the tintinnid barcode a7cbc was predicted by temperature with a negative (−0.20) regression coefficient, followed by oxygen (0.23) and density (0.18). B61a7 (tintinnid) was explained by chlorophyll (−0.18), nitrate (−0.18), and phosphate (−0.16) (Table S6). F2f8b (diatom) and a7cbc (tintinnid), the most abundant barcodes of each partner, showed opposite signs with respect to the majority of abiotic predictors, yet the organisms were paired together in similar samples. Consequently, these results suggest that conditions favoring one partner are opposite than for the other, and that other factors impact the distribution of the consortia, such as biotic interactions. Moreover, the consortia reported in this study occurred in nutrient-replete regions (Figure S4), illustrated by the higher nitrate concentrations (Welch two sample t-test *p-*value* < *0.05), contrary to other symbiotic partnerships in the ocean that often occur in oligotrophic conditions [7]. For example, Station TARA_066 is located in the Benguela Current, station TARA_070 is situated at the limit of the nitrate plume originating from the Benguela Current flowing northwest, Stations TARA_102, 106, and 109 are in the Peru Current, Station TARA_124 benefits from the island mass effect of the Marquesas archipelago, and Station TARA_139 is located in the North Pacific Equatorial Countercurrent. Taken together, these data suggest that access to nutrients is not the primary benefit for the diatoms in these consortia. However, diatoms use nutrients from the surface layer and assimilate them through photosynthesis, which can lead to the production of exudates of particulate matter that tintinnids can potentially benefit from directly or indirectly [54].

To explore biotic factors that may influence the interaction, we used the metabarcode data to examine the occurrence of the consortia with tintinnid competitors (oligotrichs) and potential predators (copepods), both known to impact tintinnid distribution [55], as well as to prey on diatoms. The total barcode abundance of oligotrichs and copepods was assessed in 126 stations sampled during the *Tara* Oceans expedition (Figure S5a,b). Barcode abundance of oligotrichs did not display an overlapping pattern with the diatom–tintinnid association. However, significantly higher copepod abundance was found in samples in which the association was observed (Welch two sample *t*-test *p-*value < 0.05), suggesting that high predation pressure may favor occurrence of the consortia. Flow cytometry counts of bacteria and pico-eukaryotes, potential prey for the tintinnids, were not significantly different in stations where the consortia were found (Figure S5c). Although tintinnids can feed on diatoms [27, 56], most of the tintinnids involved in the consortia are characterized by small lorica oral diameters suggesting that small prey items of pico- and nano-sizes are likely to be their preferred prey [57] and could potentially benefit from increased abundance of bacteria and nanoflagellate bacteriovores in the diatom phycosphere. As a complementary non-targeted approach, we searched for the tintinnid and diatom V9 sequences within a global ocean co-occurrence network [58]. Although this revealed the presence of the tintinnid a7cbc sequence, it did not co-occur with the relevant diatom species (see Supplementary Table S7 and Figure S6).

**Fig. 6:**
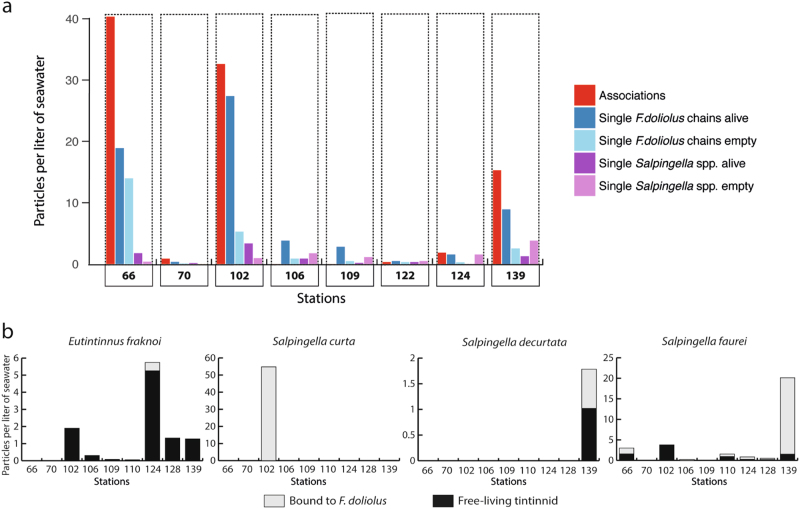
Quantification of diatom–tintinnid consortia across the ocean based on observations of net samples and converted in consortia/L of seawater (20–180 μm fraction). **a** CLSM counts of both partners and their physiological state in samples from eight *Tara* Oceans stations. **b** Counts from light microscopy observations. Four different species of tintinnids displaying associations with *F. doliolus* were counted across nine different formol-fixed samples in which the association was the most abundant (stations in the South and the North Pacific as well as the Benguela Current). The abundance of the four species per liter of seawater are shown, depending on whether they are bound to *F. doliolus* or not

Finally, the ecological importance of the diatom–tintinnid association was quantified through actual cell counts in different stations using the 20–180 µm size fractions by CLSM and light microscopy (Fig. 6) and lugol-fixed samples (Supplementary Information), allowing us to evaluate the prevalence of free-living and associated partners, as well as quantification at species level. Up to 700 diatom–tintinnid consortia per ml of net sample were enumerated, equivalent to ~40 to 50 consortia/L of seawater in samples from Station TARA_066 in the Benguela Current and TARA_102 in the upwelling Peruvian west coastal Pacific current (Figs. 6a, b). Diatom-associated *Salpingella* represented up to 95% of all *Salpingella* lorica (Figure S7) and constituted over 50% of the tintinnid community (Table S8). Conversely, over 74% of *F. doliolus* chains present in a sample could be associated to tintinnids, indicating that when both partners co-occur the association is dominant as compared with the abundances of free partners. Notwithstanding, we also found some stations in which both partners were free living. For example, in Station TARA_137 we found free *Salpingella* sp. in the absence of *F. doliolus* (data not shown). In Stations TARA_106 and TARA_109, we found low abundances of *F. doliolus* (<10 chains/L) and *Salpingella* sp., yet very few associations were detected (Fig. 6).

## Conclusions

Our study investigates the epibiotic association between the diatom *F. doliolus* and tintinnid ciliates, revealing the widespread occurrence, ecological importance, and morphogenetic characteristics of the *Fragilariopsis–Salpingella* consortia, as well as reporting five new tintinnid genera involved in the interaction.

This study is significant because it reveals that *F. doliolus* possesses a unique morphological feature, being able to form barrel-shaped chains enabling dynamic interactions with tintinnids. The species is highly abundant and distributed worldwide, and also displays biogeographic structuring evidenced by the first molecular data available for this species. Moreover, analysis of the *Salpingella* sp. brings to light two major haplotypes that are not related to geographic separation yet correspond to phenotypic features. The combination of morphological and molecular studies reveals the presence of viable diatom and tintinnid cells in a motile consortium, a widespread distribution in nutrient-replete and predator-rich conditions, as well as a long-term nonspecific stability. These observations support the hypothesis of a mutualistic symbiosis, wherein diatoms would acquire increased motility and tintinnids benefit from silicification through increased protection. Our study not only highlights an abundant and ubiquitous marine microbial interaction by integrating data from the single cell to the global ocean, but also explores potential benefits underlying such an association that further studies will help elucidate.

## Electronic supplementary material

Video S1

Video S2

Video S3

Video S4

Supplementary Information

Supplementary Figures

Table S1

Table S2

Table S3

Table S4

Table S5

Table S6

Table S7

Table S8
