# Supplementary material for: The epibiotic life of the cosmopolitan diatom Fragilariopsis doliolus on heterotrophic ciliates in the open ocean
Source: ISME J. 2018 Jan 18;12(4):1094–108. doi: 10.1038/s41396-017-0029-1 (PMC5864193; doi:10.1038/s41396-017-0029-1)
Supplement: Supplementary file 5 — Supplementary Information [file 41396_2017_29_MOESM5_ESM.pdf]

Supplementary Information

Supplementary Methods

First isolation and non-targeted PCR amplification of the V4-V9 18S rDNA subregion. The first isolation was performed in the lab using formaldehyde-glutaraldehyde fixed samples from Station TARA_066 using an inverted microscope on bright field mode (Zeiss Axiovert 135) and a bulb-operated hand aspirator. Individual consortia were isolated based on morphological traits of the barrel-shaped diatom, which was easy to recognize and highly specific of this diatom species. However, we could not properly distinguish the different *Salpingella* species and therefore considered all consortia involving *Fragilariopsis doliolus* with *Salpingella*. Individual consortia only contained one tintinnid, but the diatom barrel displayed (on average) between 3-10 cells.

Individual consortia were rinsed two to three times in a minimum volume of sterile artificial sea water, immersed in 300 µL of Tissue and Cell lysis buffer from MasterPure DNA and RNA purification kit (Epicenter), and stored at -20 °C. DNA extraction was performed following the protocol of the MasterPure DNA and RNA purification kit (Epicenter). Polymerase chain reactions (PCR) using universal-eukaryotic primers V4F 5'- CCAGCASCTGCGGTAATTC – 3' and 1510R 5'- CCTTCYGCAGGTTCACCTAC- 3' were performed on total DNA extracts. Working on isolated and rinsed consortia decreased the chances of amplifying organisms other than the diatom-tintinnid consortia.

PCR amplifications were conducted with the Phusion High-Fidelity DNA Polymerase (ThermoFisher Scientific). The PCR mixture (25 µL final volume) contained 2 µL of DNA, 0.5 µM (final concentration) of each primer, 3% of dimethyl sulfoxide, 200 µM dNTPs and 5X Phusion HF Buffer. Amplifications were conducted with the following PCR program: initial denaturation step at 98 °C for 30 s, followed by 28 cycles of 10 s at 98 °C, 45 s at 55 °C, 15 s at 72 °C and final elongation step at 72 °C. Amplicons were purified using the Nucleospin Gel and PCR Clean-up (Macherey-Nagel) with two NT3 rinsing due to the small quantities of DNA. In order to recover the partial 18S rDNA of both partners in the consortium, a cloning approach was adopted: purification was followed by a poly-A tailing reaction using GoTaq DNA Polymerase (Promega) and ligated in pGEM-T Easy Vector Systems (Promega). The product was cloned using chemically competent *Escherichia coli* cells, colonies were selected with blue white IPTG/Xgal and length of the insert was checked by PCR before picking. Plasmids were purified with the Plasmid DNA purification kit (Macherey-Nagel) and double-end sequenced by GATC Sanger sequencing.

Cloning of 18S rDNA amplified from single diatom chains often revealed intra-individual genetic micro-diversity, with at least three different copies of 18S containing 28 nucleotide differences over a 1,246 bp alignment. The three 18S copies also happened to be different at the level of the V9 subregion (Table S2) with a maximum distance of four nucleotide differences between two V9 subregions. A single 18S rDNA tintinnid sequence showed at least four different copies of 18S displaying four polymorphic sites. However, three of these copies had an identical V9 sequence.

Targeted PCR amplification of small subunit rDNA genes, internal transcribed spacers ITS1 and ITS2, 5.8S and 28S rDNA genes. PCR mixtures (25 µl final volume) contained 2 µl of DNA, 0.35 µM (final concentration) of each primer, 3% of dimethyl sulfoxide and 2× of GC buffer Phusion MasterMix (Finnzymes). Amplifications were conducted in a PCR thermocycler (Applied Biosystems) with the following PCR program: initial denaturation step at 98 °C for 30 s, followed by 37 cycles of 10 s at 98 °C, 30 s at the annealing temperature of 50 °C 30 s at 72 °C and final elongation step at 72 °C for 10 min. Amplicons were purified using Nucleospin Gel and PCR Clean-up (Macherey-Nagel), reamplified using internal primers to cover the 18S, ITS1&2, 5.8S and partial 28S then Sanger sequenced using the ABI-PRISM Big Dye Terminator Sequencing kit (Applied Biosystems).

Phylogenetic analysis. For the tintinnids, contigs of the amplicons were obtained and two matrices of 18S rDNA and ITS+5.8S+28S rDNA were built that included reference sequences from (Bachy, 2012b). For diatoms, similar matrices were built including reference sequences from BLAST top hits in GenBank and reference sequences from (Theriot, 2010). The four matrices were completed with appropriate outgroup sequences, and aligned using MAFFT version 7 (Kazutaka, 2013). Sequences were trimmed using Gblocks (Talavera, 2007) as implemented in SeaView (Gouy, 2010) allowing for smaller final blocks, gap positions within the final block, and less strict flanking positions, with various degrees of stringent set selection. JmodelTest (Darriba, 2012) was used to determine the best model of nucleotide substitution for each matrix. The general time-reversible model with gamma distribution of rate variation (GTR+G) was selected for the diatom ITS+5.8S+28S, and the tintinnid 18S tree which are the best markers, respectively, for diatom and ciliates. Phylogenetic inference was performed using PhyML 3.0 (Guindon, 2010) and robustness of topologies assessed by performing 1,000 bootstraps. Bayesian inference analyses were carried out on the tintinnid 18S rDNA tree, with the program MrBayes (Huelsenbeck, 2001), with two independent runs and 10^6 generations per run. After checking convergence (maximum difference between all bipartitions <0.01) and eliminating the first 3,500 trees (burn-in), a consensus tree was constructed sampling every 100 trees. Trees were visualized and edited using FigTree v.1.4.2. For ITS+5.8S+28S rDNA, a statistical parsimony network was constructed with TCS software (95% connection limit, 10 or less connection steps, gaps considered as 5th state) (Clement *et al.*, 2000), and visualised using TCS. All alignments used for phylogenetic inference are available in Table S5.

Morphological quantification of the consortium. Quantitative microscopy was carried out on samples prepared as described in (Colin *et al.*, 2017), fixed in 1% formaldehyde-0.25% glutaraldehyde. Images were processed for automated cell detection in the sample. Each detected object in the sample preparation was visually checked based on specific 3D-multicolor thumbnails that are generated by the processing pipeline. *F. doliolus* and the tintinnid species that are involved in the association were classified according to free and

associated forms and viability status (presence of cell body inside the frustule or lorica) were quantified. Quantitation was normalized based on the total sample volumes (see Pesant *et al.*, 2015), and the abundances were then determined as cells / L of seawater, thus allowing sample abundance comparison.

For comparison, quantification was also performed by light microscopy. Particles per liter of seawater counts were determined from 4% formaldehyde fixed samples as shown in Figure 6. Aliquots of formaldehyde-fixed plankton net material (0.5 - 2.0 ml) were examined in plankton settling chambers using an Inverted Olympus microscope (model IX71) equipped with DIC optics at 200x total magnification. Data were compared to cell/mL counts of total tintinnids encountered in other *Tara* Oceans samples as shown in Figure S7 and raw counts are available in Table S8.

It is noteworthy that cell quantification differed based on the fixation procedure; consortia counts between lugol- and formaldehyde-fixed samples of plankton net material showed high discrepancies, in that the association was typically much less abundant in acidic lugol, which is not effective in preserving the association. Fixative bias is well documented for tintinnid cells (Modigh, 2005), but appears also to affect the detection of biotic consortia. Along with the absence of mechanical structures for attachment, this suggests that the association is maintained by the presence of adhesive extracellular polymeric substances (EPS) secreted by the ciliate or the diatom, maybe sensitive to acidic fixators.

<u>Taxonomic features for species delineation.</u> Identification of the diatom species was unambiguous according to the "Identifying Marine Phytoplankton" procedures (see Tomas, 1997; page 305) and based on the location and motifs of the poroids, the raphe system, and the valve shape. It is readily identified due to the unique shape of the ribbons and the valve outline.

Tintinnid identifications were made based on lorica characteristics and dimensions following standard taxonomic monographs (Kofoid, 1929; Kofoid, 1939; Hada, 1938). Below we provide a description of the morphological features of the tintinnid loricas upon which our species identifications were based and references for the original species descriptions :

- **Genus *Amphorellopsis***: Lorica material hyaline, no obvious secondary structure. Aboral end closed and pointed. Lorica with folds or blades. Specimen in figure 2 (e) is perhaps a collapsed (flattened) *A. turbinea*, described as having an lorica oral opening diameter of about 20 µm, broad blade fins running the length of the lorica, 70 µm in length (Kofoid & Campbell 1929).
- **Genus *Amphorides***: Lorica material hyaline, no secondary structure obvious. Overall shape is vase-like with longitudinal ridges or fin with a truncated aboral end. Oral margin smooth. *A. laackmanni* (Jorgensen 1924)*:* Urn-shaped with slight expansion at the oral end. Lorica oral opening diameter of about 20 µm. 4-6 ridges or fins run the length of the lorica. Truncated oral end of about 2 µm diameter. Overall lorica length of about 70 µm.

- **Genus *Ascampbelliella***: Small cup or goblet -shaped lorica with the lorica oral opening separated from the rest of the lorica by a trough between it and a second, and in some species a third oral collar. Lorica material hyaline with no obvious secondary structure. *A. tortulata* (Jorgensen 1924): Bell-shaped lorica with a single secondary collar, slighlty pointed aboral end. Lorica oral opening diamter of about 22 µm, lorica length of 30 µm
- **Genus *Dictyocysta***: Lorica bell-shaped. Collar of lorica oral opening cylindrical with a single or double row of windows. *D. mitra* (Haeckel 1873): Entire lorica a lattice-work of windows with window size diminishing toward the slightly pointed aboral end of the lorica. Lorica oral opening diameter about 50 µm, lorica length about 70 µm.
- **Genus *Eutintinnus***: Lorica material hyaline, no secondary structure obvious. Overall shape is tubular, open at both ends. Specimens in Figure 2 (f,g) most closely resemble *E. fraknoii* (Daday 1887) or *E. lusus-undae* (Entz 1885). As stated by Jorgensen (1924) the two forms are '...nearly allied and so closely connected by intermediate forms that it is difficult to draw any line of seperation between them".
- **Genus *Protorhabdonella***: Lorica hyaline, wall with no visible secondary structure but with vertical ribs, no gutter in oral opening, aboral end closed, no aboral pedicels or knobs. Conical or chalice-shaped. *P. striatura* (Kofoid & Campbell 1929): Lorica elongate conical with 24-28 ribs, pedicel slightly differentiated. Lorica oral opening diameter of about 30 µm, lorica length 145 µm.
- **Genus *Salpingella***: Lorica material hyaline, no secondary structure. Lorica shape is elongate, overall nail-shaped. Oral opening smoooth (no teeeth or secondary structures), aboral end open. *Salpingella faurei* (Kofoid & Campbell 1929): Thin, nearly cylindrical with slight expansion of oral end. Oral margin not everted or thickened. Lorica oral opening diameter approx 10 µm. Several slighty curved ridges or fins run along the lorica and are most prominant in the lower half. In contrast to other *Salpingella species*, lorica integrity appears weak as loricas are often collapsed. Aboral end truncated with a very small aperature (1-2 µm). Overall length highly variable 50 - 100 µm. *Salpingella decurtata* (Jorgensen 1924): Lorica shape nearly cylindrical with expansion of oral end. Oral margin clearly everted outward and thickened. Lorica oral opening diameter approx 18 µm. Ridges or fins along the lorica most prominant in the lower third, aboral end truncated with an aperature of about 2 µm. Overall length highly variable 120 - 160 µm. *Salpingella curta* (Kofoid & Campbell 1929): Cylindrical overall, abruptly conical in lower quarter. Oral margin very slightly everted with slightlly thickened edge. Lorica oral opening diameter of about 14 µm (12-16 µm range). Obvious ridges in lower third of lorica. Aboral end opening of 2-3 µm. Overall lorica length 75-125 µm.

Co-occurrence network analysis. The five different V9 barcodes corresponding to isolated organisms available in Table S3 were searched for in the *Tara* Oceans co-occurrence network published in Lima-Mendez et al., 2015, in order to characterize the organisms with which the barcodes were detected to be significantly correlated to, both positively and negatively. The co-occurrence network is shown in Figure S4 and correlation values in Table S7.

One tintinnid barcode that was present was a7cbc (Figure S6), which co-occurred significantly with *Eutintinnus* (found in association with *F. doliolus* in our samples) and *Amphorides* ciliates. Conversely, two diatom barcodes, assigned to Bacillariophyta_X and *Ditylum*, positively correlated with *Salpingella* but were highly divergent from *F. doliolus* based on the V9 sequences (87% ID). We therefore conclude that a co-occurrence network built at such a large scale was unable to recover the biotic consortium reported here.

Supplementary Figures

Figure S1: Confocal laser scanning microscopy of *Amphorides laackmanni* associated with *F. doliolus* in Station TARA_102. (a) Contact zone (b-c) Close up views of the contact zone in (a). Scale bar = 10 µm.

Figure S2 : Location of *Tara* Oceans stations. Station TARA_066 is framed in red**,** located in the Benguela Current, in which the diatom-tintinnid interaction was initially observed, in surface samples from the 20-180 micron size fraction, preserved in glutaraldehyde-paraformaldehyde. Other stations in which the interaction was observed are framed in blue. Image adapted from N. Le Bescot.

Figure S3 : Spatial distribution of the V9 sequences obtained by single consortia sequencing of the diatom – tintinnid consortia, across the 126 *Tara* Oceans stations in surface samples. (a) Merged view of the abundance of the four amplified barcodes, including the two most abundant ones f2f8b (diatom) and a7cbc (tintinnid) in three size fractions, in different ocean provinces. The abundances of diatom and tintinnid were positively correlated (Spearman *rho* = 0.37, *pval* < 2.2e-16). Stations in which the consortia were found are marked in red on the x-axis and indicated with an arrow. (b) Abundance of the diatom 53cf4 sequence, assigned as Raphid-pennate_X+sp. in *Tara* Oceans samples, initially obtained from the diatom sequenced from Station TARA_102 (see Figure S2). (c) Abundance of the tintinnid deb2a assigned to Choreotrichia_XX+sp in *Tara* Oceans samples. Absolute abundance was transformed according to the log(Abundance+1) formula with low to high abundances corresponding to small and big bubbles, respectively.

Figure S4 : Heatmap of *Tara* Oceans contextual environmental data in surface samples of 20-180 micron fractions, based on partial least square regression predictors and derived from 126 stations. Values of each environmental variable were standardized into a range 0 to 1 and plotted using the pheatmap function in R package «pheatmap ». Stations are indicated on the x axis. Stations in which the diatom-consortia were observed are framed within black rectangles and displayed on average a higher nitrate concentration (Welch Two Sample t-test *pvalue* < 0.05).

Figure S5 : Spatial distribution of tintinnid predators, competitors and prey in the *Tara* Oceans data. (a) Absolute V9 abundance of all copepod barcodes merged together in surface samples in 20-180 and 180-2000 micron size fractions, extracted from de Vargas *et al.*, 2015. The copepod abundance was higher in the samples in which the association was observed (Welch Two Sample t-test *pvalue* < 0.05)(b) V9 Abundance of all Oligotrich barcodes grouped together in surface samples in 20-180 and 180-2000 micron size fractions, extracted from de Vargas *et al.*, 2015. (c) Flow cytometer-based counts of bacteria (in red) and picoeukaryotes (in blue), in cells/mL. Extracted from Sunagawa *et al.*, 2015, Supplementary Table W8.

Stations in which the consortia were found are marked in red on the x-axis and indicated with an arrow.

Figure S6: Co-occurrence network of the tintinnid-derived V9 sequence A7cbc. Co-occurrence network extracted from the *Tara* Oceans interactome (Lima-Mendez *et al.,* 2015). Each node represents a unique barcode and is colored and named by its taxonomic group. Red edges represent mutual exclusions, and green edges represent barcodes that are significantly positively correlated. The extended list is available in Table S7.

Figure S7 : Tintinnid cell counts in *Tara* Oceans stations based on quantification per mL of 4% formaldehyde-fixed samples in 20-180 micron size fraction. Total tintinnid counts in black, diatom-associated *Salpingella* species in light grey, and single *Salpingella* species in grey.

Supplementary Tables

Table S1: Reference of the *Tara* Oceans samples used in the study used for individual consortia isolation and sequencing, and consortia quantification. Genbank and ENA accession numbers of sequences obtained in this paper are available.

Table S2: Summary of obtained V9 sequences for single isolated interactions and universal PCR amplification from CLSM surface samples (1% formaldehyde -0.25% glutaraldehyde), 20-180 micron fraction.

Table S3: Abundance of V9 barcodes in the global *Tara* Oceans dataset, corresponding to sequences of amplified diatom and tintinnid consortia.

Table S4: Primers used for amplification and sequencing, as well as table with the combination of tintinnid and diatom V9 sequences used to search for specific pairs.

Table S5 : Alignments used for phylogenetic analysis and the illustration of single cell 18S microheterogeneity with one diatom and one tintinnid single cell from consortia TI_40.

Table S6: Partial Least Square Analysis with regression coefficients and environmental parameters after range transformation in 126 stations used as predictors and V9 abundance of the five ribotypes after Hellinger transformation used as response.

Table S7: Subset of the *Tara* Oceans interactome (Lima-Mendez *et al*, *2015*) for ribotype a7cbc.

Table S8: Tintinnid cell counts in *Tara* Oceans samples based on 4% formaldehyde - and lugol-fixed samples. Numbers are given in cells per mL of fixed samples. Concentration factors are provided for conversion from cells/mL of fixed samples in cells/L of seawater.

References for Supplementary Information

Daday, E. von 1887. Monographie der Familie der Tintinnodeen. Mittheilungen aus der Zool. Station zu Neapel, 7: 473-591

Entz, G. 1885. Zur nähren Kenntnis der Tintinnoden. Mittheilungen aus der Zoologischen Station zu Neapel, 6:185-216.

Gouy M, Guindon S, and Gascuel O. (2009). SeaView Version 4: a multiplatform graphical user interface for sequence alignment and phylogenetic tree building. *Molecular Biology and Evolution* 27.2: 221-24.

Haeckel, E. 1873. Ueber einige pelagische Infusorien. Jenaische Zeitschrift für Medizin und Naturwissenschaft, 7:561-568, Plates 27-28

Jörgensen, E. 1924. Mediterranean Tintinnids. Report on the Danish Oceanographical Expeditions 1908-10 to the Mediterranean and adjacent Seas. 2 J.3. (Biology):1-110

Kofoid CA, Campbell AS. (1929) A Conspectus of the Marine and Freshwater Ciliata Belonging to the suborder Tintinnoinea, with Despcriptions of New Species Principally from the Agassiz Expedition to the Eastern Tropical Pacific 1904-1905. University of California Publications in Zoology 34, 1-403.

Kofoid CA, Campbell AS. (1939) Reports on the scientific results of the expedition to the Eastern Tropical Pacific, in charge to Alexander Agassiz, by U.S. Fish Commission Steamer "Albatross". from October 1904 to March 1905, Lieut. Commander L.M. Garrett, U.S.N. commanding. 37. The Ciliata: The Tintinnoinea. Bulletin of the Museum of Comparative Zoology, Harvard 84, 1-473.

Hada Y. (1938) Studies on the Tintinnoinea from the Western Tropical Pacific. Journal of the Faculty of Science Hokkaido Imperial University, Series 6, Zoology, 6: 87-190.

Modigh M, Castaldo S. (2005). Effects of fixatives on ciliates as related to cell size. *Journal of Plankton Research* 27.8: 845-49.

Sunagawa S, Coelho LP, Chaffron S, Kultima JR, Labadie K, Salazar G *et al*. (2015). Structure and function of the global ocean microbiome. *Science*, *348* : 6237.

Tomas, C.R., (ed.) 1997. Identifying marine phytoplankton. Academic press.
