# Supplementary material for: The epibiotic life of the cosmopolitan diatom Fragilariopsis doliolus on heterotrophic ciliates in the open ocean
Source: ISME J. 2018 Jan 18;12(4):1094–108. doi: 10.1038/s41396-017-0029-1 (PMC5864193; doi:10.1038/s41396-017-0029-1)
Supplement: Supplementary file 6 — Supplementary Figures [file 41396_2017_29_MOESM6_ESM.pdf]

Figure S1

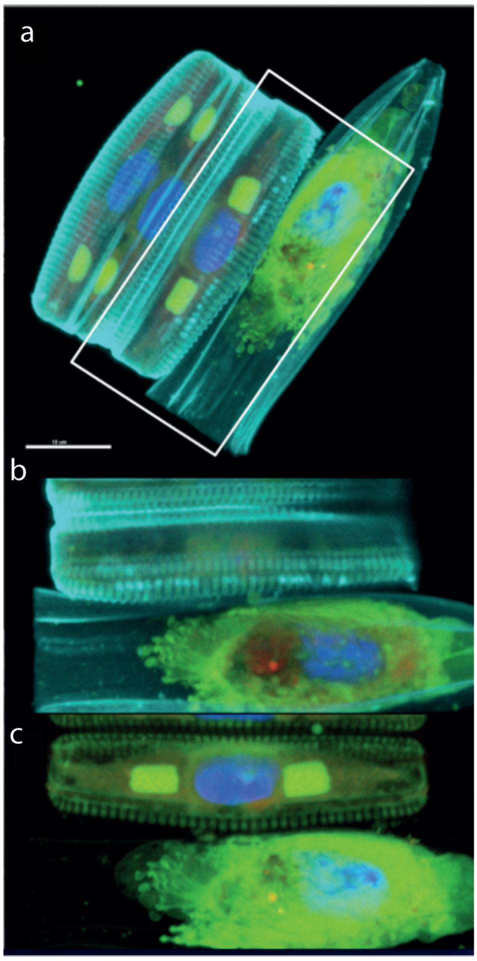

Figure S1: Confocal laser scanning microscopy of *Amphorides laackmanni* associated with *F. doliolus* in Station TARA_102. (a) Contact zone (b-c) Close up views of the contact zone in (a). Scale bar = 10 µm.

Figure S2

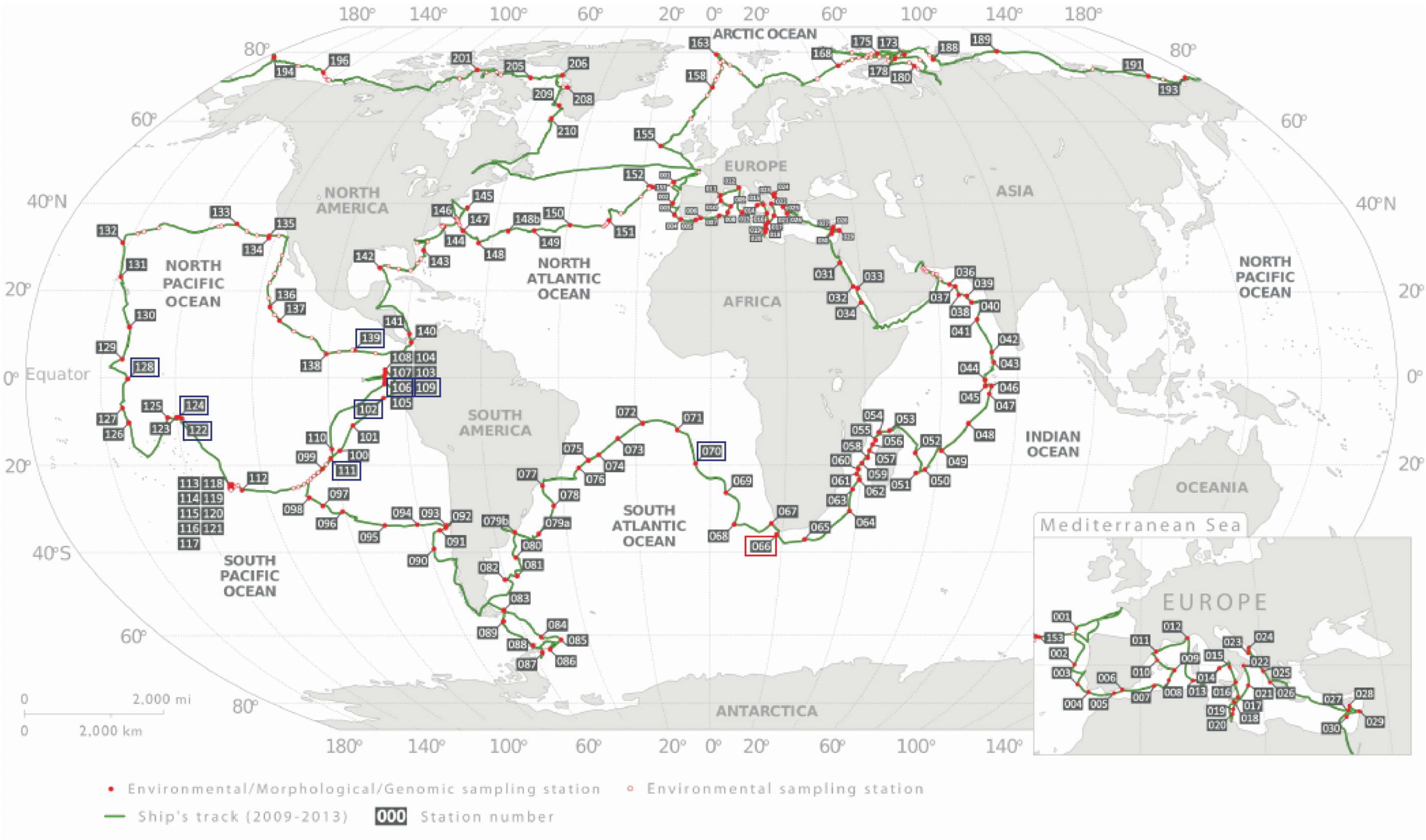

Figure S2 : Location of *Tara* Oceans stations. Station TARA_066 is framed in red**,** located in the Benguela Current, in which the diatom-tintinnid interaction was initially observed, in surface samples from the 20-180 micron size fraction, preserved in glutaraldehyde-paraformaldehyde. Other stations in which the interaction was observed are framed in blue. Image adapted from N. Le Bescot.

Figure S3

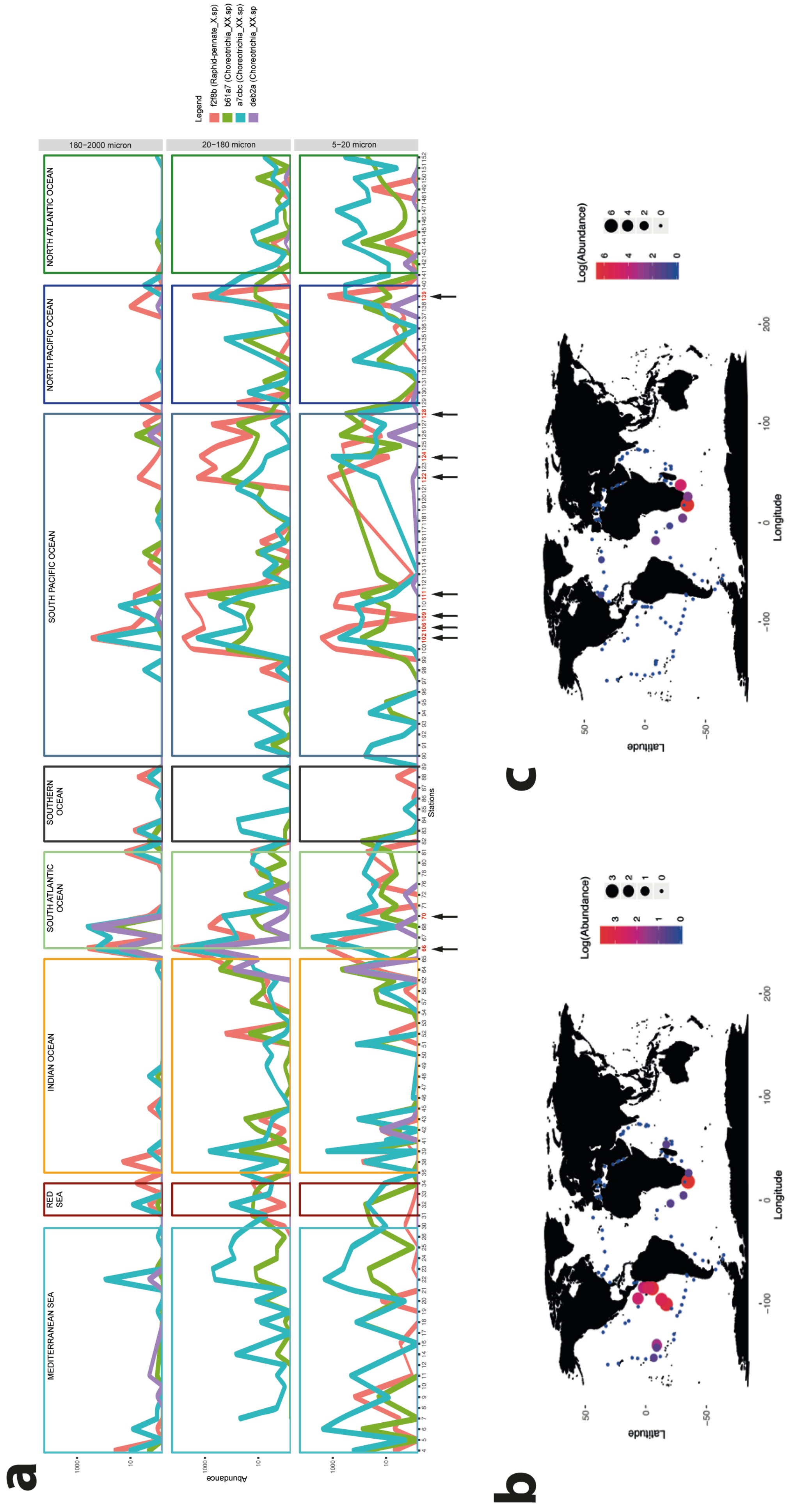
a
b
c
180–2000 micron
20–180 micron
5–20 micron
Legend
f2f8b (Raphid-pennate_X sp)
b61a7 (Choreotrichia_XX sp)
a7cbc (Choreotrichia_XX sp)
deb2a (Choreotrichia_XX sp)
MEDITERRANEAN SEA
RED SEA
INDIAN OCEAN
SOUTH ATLANTIC OCEAN
SOUTHERN OCEAN
SOUTH PACIFIC OCEAN
NORTH PACIFIC OCEAN
NORTH ATLANTIC OCEAN
Abundance
Stations
Log(Abundance)
Latitude
Longitude

Figure S3 : Spatial distribution of the V9 sequences obtained by single consortia sequencing of the diatom – tintinnid consortia, across the 126 *Tara* Oceans stations in surface samples. (a) Merged view of the abundance of the four amplified barcodes, including the two most abundant ones f2f8b (diatom) and a7cbc (tintinnid) in three size fractions, in different ocean provinces. The abundances of diatom and tintinnid were positively correlated (Spearman *rho* = 0.37, *pval* < 2.2e-16). Stations in which the consortia were found are marked in red on the x-axis and indicated with an arrow. (b) Abundance of the diatom 53cf4 sequence, assigned as Raphid-pennate_X+sp. in *Tara* Oceans samples, initially obtained from the diatom sequenced from Station TARA_102 (see Figure S2). (c) Abundance of the tintinnid deb2a assigned to Choreotrichia_XX+sp in *Tara* Oceans samples. Absolute abundance was transformed according to the log(Abundance+1) formula with low to high abundances corresponding to small and big bubbles, respectively.

Figure S4

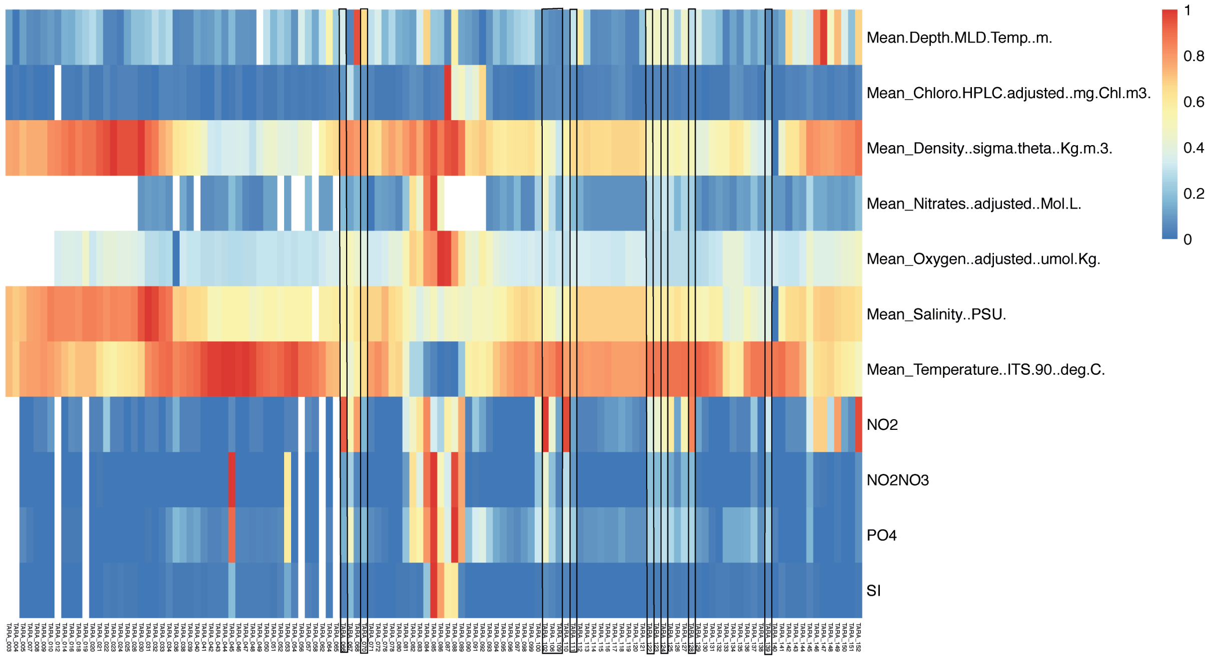

Figure S4 : Heatmap of *Tara* Oceans contextual environmental data in surface samples of 20-180 micron fractions, based on partial least square predictors and derived from 126 stations. Values of each environmental variable were standardized into a range 0 to 1 and plotted using the pheatmap function in R package «pheatmap ». Stations are indicated on the x axis. Stations in which the diatom-consortia were observed are framed within black rectangles and displayed on average a higher nitrate concentration (Welch Two Sample t-test *pvalue* < 0.05).

Figure S5

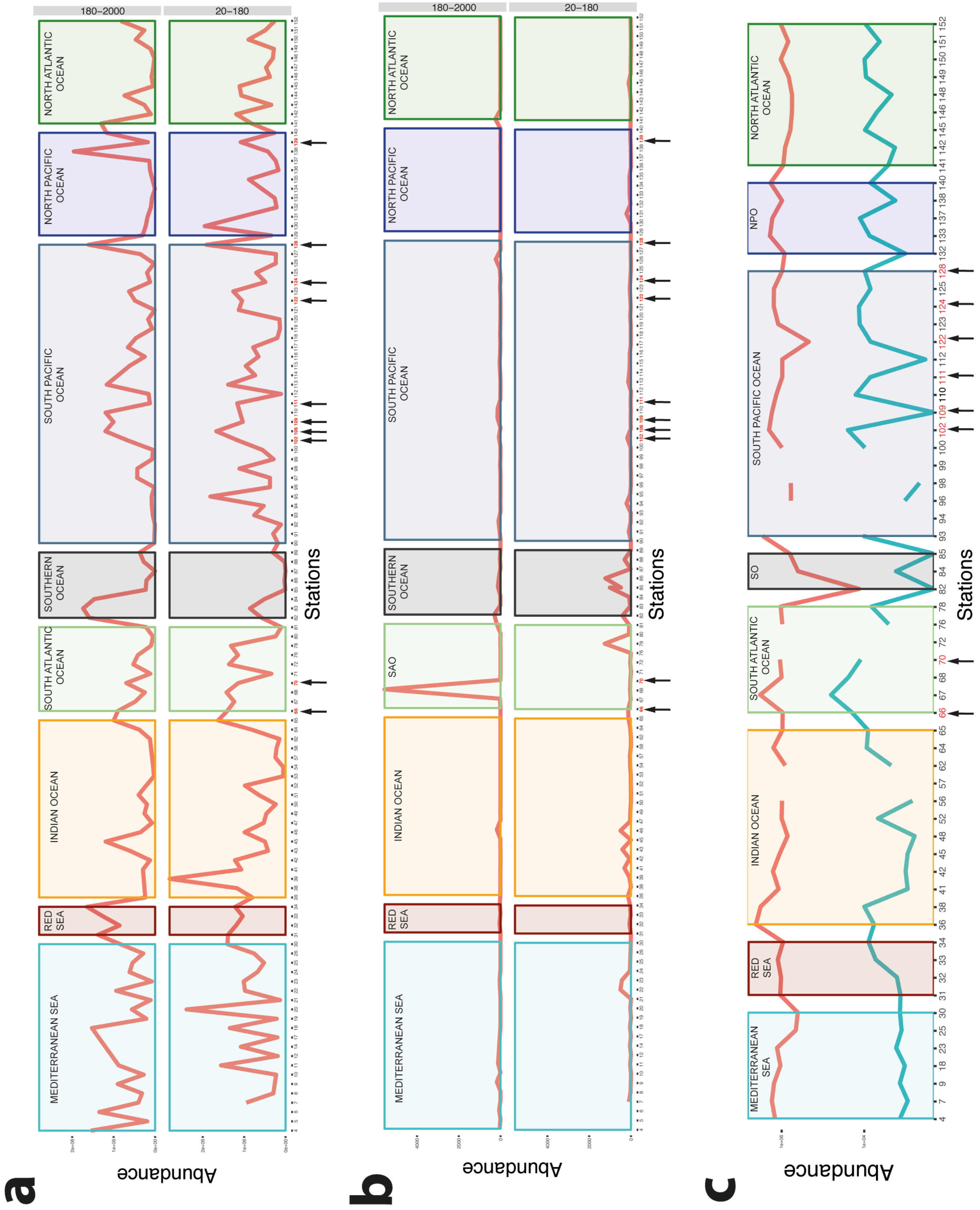

a
b
c
Abundance
Stations
180–2000
20–180
MEDITERRANEAN SEA
RED SEA
INDIAN OCEAN
SOUTH ATLANTIC OCEAN
SOUTHERN OCEAN
SOUTH PACIFIC OCEAN
NORTH PACIFIC OCEAN
NORTH ATLANTIC OCEAN
SAO
SO
NPO

Figure S5 : Spatial distribution of tintinnid predators, competitors and prey in the *Tara* Oceans data. (a) Absolute V9 abundance of all copepod barcodes merged together in surface samples in 20-180 and 180-2000 micron size fractions, extracted from de Vargas *et al.*, 2015. The copepod abundance was higher in the samples in which the association was observed (Welch Two Sample t-test *pvalue* < 0.05)(b) V9 Abundance of all Oligotrich barcodes grouped together in surface samples in 20-180 and 180-2000 micron size fractions, extracted from de Vargas *et al.*, 2015. (c) Flow cytometer-based counts of bacteria (in red) and picoeukaryotes (in blue), in cells/mL. Extracted from Sunagawa *et al.*, 2015, Supplementary Table W8. Stations in which the consortia were found are marked in red on the x-axis and indicated with an arrow.

Figure S6

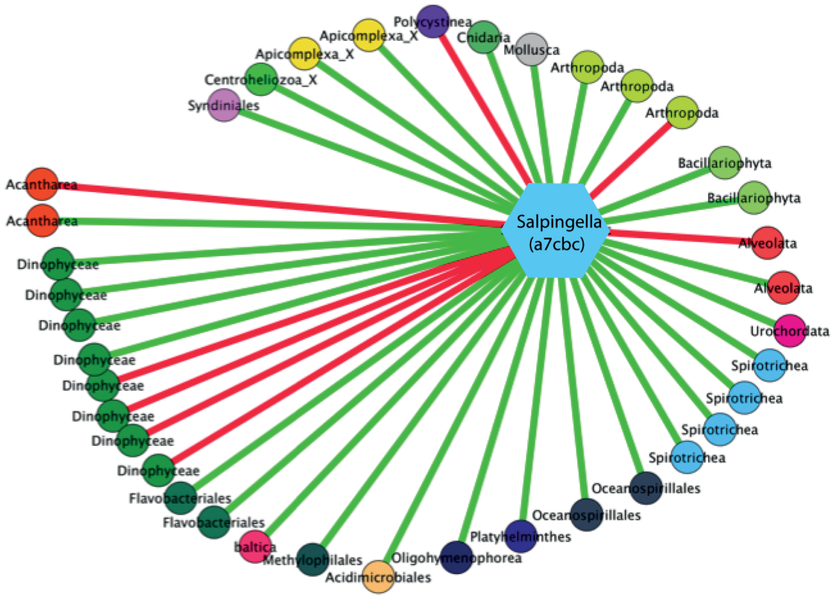

Figure S6: Co-occurrence network of the tintinnid-derived V9 sequence A7cbc. Co-occurrence network extracted from the *Tara* Oceans interactome (Lima-Mendez *et al.,* 2015). Each node represents a unique barcode and is colored and named by its taxonomic group. Red edges represent mutual exclusions, and green edges represent barcodes that are significantly positively correlated. The extended list is available in Table S7.

Figure S7

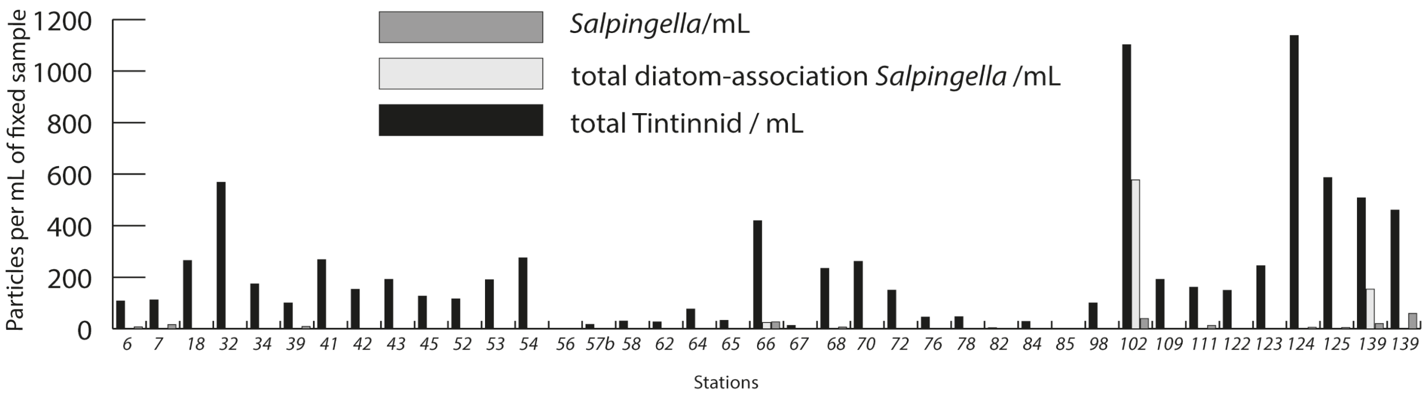

Figure S7 : Tintinnid cell counts in *Tara* Oceans stations based on quantification per mL of 4% formaldehyde-fixed samples in 20-180 micron size fraction. Total tintinnid counts in black, diatom-associated *Salpingella* species in light grey, and single *Salpingella* species in grey.
